# Genome-based reclassification of Alkalispirochaeta sphaeroplastigenens (Vishnuvardhan Reddy et al. 2013) Sravanthi et al. 2016 as a later heterotypic synonym of Alkalispirochaeta alkalica (Zhilina et al. 1996) Sravanthi et al. 2016

**DOI:** 10.1099/ijsem.0.007205

**Published:** 2026-06-22

**Authors:** Michaela M. Scranton, Richard W. McLaughlin, Mostafa S. Elshahed, Paul A. Lawson, Samuel L. Miller

**Affiliations:** 1 School of Liberal Arts and Sciences, Gateway Technical College, Kenosha, WI 53144, USA; 2 Department of Microbiology and Molecular Genetics, Oklahoma State University, Stillwater, OK 74074, USA; 3 School of Biological Sciences, University of Oklahoma, Norman, OK 73019, USA

**Keywords:** *Alkalispirochaeta sphaeroplastigenens*, *Alkalispirochaeta alkalica*, Heterotypic synonym

## Abstract

An initial comparison of 16S rRNA gene sequences between *Alkalispirochaeta alkalica* Z-7491^T^ and other members of the genus *Alkalispirochaeta* revealed ≥99.4% sequence similarity, suggesting their close relatedness and the possibility that some members are in fact the same species. The genus *Alkalispirochaeta* includes five species with validly published names. *A. alkalica* Z-7491^T^ (=ATCC 700262^T^=DSM 8900^T^) and *Alkalispirochaeta sphaeroplastigenens* JC133^T^ (=KCTC 15220^T^=NBRC 109056^T^) were both isolated from alkaline lakes (Lake Magadi in Kenya and Lonar Lake in India), respectively. The present study used whole-genome data to clarify the taxonomic assignment of these two closely related *Alkalispirochaeta* species. *A. alkalica* Z-7491^T^ and *A. sphaeroplastigenens* JC133^T^ share similar phenotypic and chemotaxonomic characteristics. They are Gram-stain-negative, motile, helical-shaped bacteria that require sodium for growth and grow optimally under alkaliphilic and mesophilic conditions, and their main cellular fatty acid is C_18 : 1_ ω7c. Overall genomic relatedness indices analyses indicated average nucleotide identity and digital DNA–DNA hybridization values >95.0% and >70.0%, respectively. These values exceed thresholds currently accepted for bacterial species delineation. Further, these taxa cluster together within the genus *Alkalispirochaeta* in both the 16S rRNA gene phylogenetic tree and the core-genome phylogenomic tree. Based on the combined evidence and the earliest validly published names, priority is given to *Alkalispirochaeta alkalica* (Zhilina *et al*. 1996) Sravanthi *et al*. 2016. *Alkalispirochaeta sphaeroplastigenens* (Vishnuvardhan Reddy *et al*. 2013) Sravanthi *et al*. 2016 is proposed to be a later heterotypic synonym of *Alkalispirochaeta alkalica* (Zhilina *et al*. 1996) Sravanthi *et al*. 2016.

 The genus *Alkalispirochaeta* was proposed in 2016 by Sravanthi *et al*. [1], and currently includes five species with validly published names (https://doi.org/10.83108/rn.518935, February 2026). Members of this genus have been isolated from alkaline lakes [2,4], the gut of cockroaches [1] and the termite gut [5]. *Alkalispirochaeta alkalica*, the type species of the genus *Alkalispirochaeta*, was isolated from an alkaline lake (Lake Magadi, Kenya) [2]. Another *Alkalispirochaeta* strain, *Alkalispirochaeta sphaeroplastigenens* was also isolated from an alkaline lake (Lonar Lake, India) [4]. Both strains are Gram-stain-negative, motile, helical-shaped bacteria that require sodium for growth and grow optimally under alkaliphilic and mesophilic conditions. Additionally, both strains are resistant to rifampicin and kanamycin, and negative for catalase, indole production and gelatinase [2,4]. Similar chemotaxonomic profiles are also observed between these strains, with the major polar lipids being an unidentified phospholipid, phosphatidylglycerol, a glycolipid and the unidentified lipids L1, L3 and L4 [4].

Upon comparison, the 16S rRNA gene sequences of *A. alkalica* Z-7491^T^ (GenBank accession number X93927) and *A. sphaeroplastigenens* JC133^T^ (GenBank accession number HE806187) were found to be 99.5% identical (Table 1). To further confirm the relationships within the genus, the EzBioCloud server [6] was subsequently used to retrieve 16S rRNA gene sequences from members of the genus *Alkalispirochaeta* and closely related genera with validly published names. The sequences were then aligned using clustalW [7], and a phylogenetic tree was constructed in mega 12 [8] using the maximum-likelihood (ML) algorithm [9]. The close relationship was supported by the 16S rRNA gene similarity values (Table 1) and the 16S rRNA gene phylogenetic tree (Fig. 1). This result indicated a very close relationship, as the similarity value exceeded the 98.7% threshold used to assign strains to a species [10]. The Kimura two-parameter model was used to calculate genetic distances [11]. Bootstrap values were calculated using 1,000 replications [12].

**Fig. 1.**
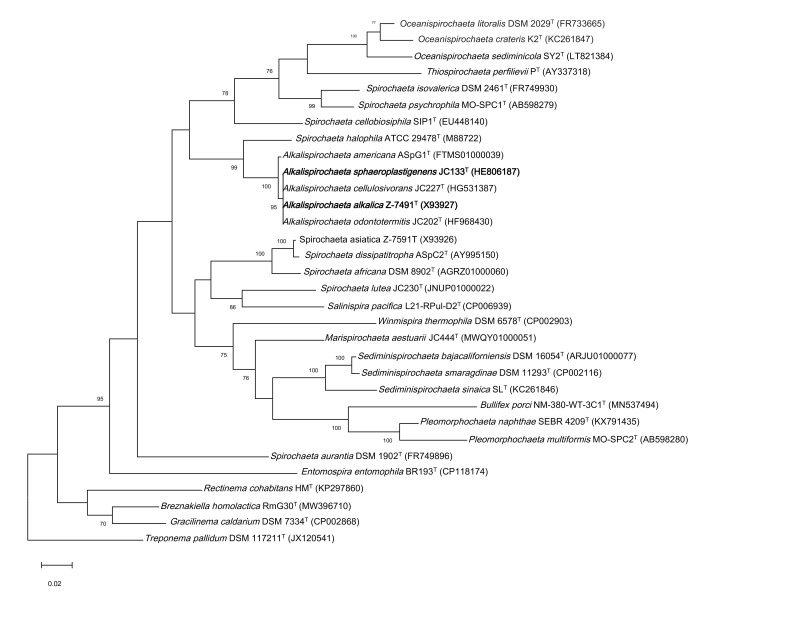
Phylogenetic tree based on 16S rRNA of *A. alkalica* Z-7491^T^ and *A. sphaeroplastigenens* JC133^T^, and close relatives. The tree was constructed using the ML method. Bootstrap values, expressed as percentages of 1,000 replications, are given at branching points. Database accession numbers are given in parentheses. Bar, 0.02 substitutions per nucleotide position.

**Table 1.** Pairwise comparison of 16S rRNA gene similarity and overall genomic relatedness indices between *A. alkalica* Z-7491^T^ and *A. sphaeroplastigenens* JC133^T^ and their close relatives

16S rRNA gene similarity (%)			
**Taxon**	**16S rRNA gene accession**	***A. alkalica***	***A. sphaeroplastigenens***
***A. alkalica***	**X93927**	**100.0**	**99.5**
***A. sphaeroplastigenens***	**HE806187**	**99.5**	**100.0**
*Alkalispirochaetaamericana*	FTMS01000039	99.3	99.0
*Alkalispirochaeta odontotermitis*	HF968430	99.8	99.9
*Alkalispirochaeta cellulosivorans*	HG531387	99.4	99.8
**dDDH (%**)			
**Taxon**	**WGS accession**	***A. alkalica***	***A. sphaeroplastigenens***
***A. alkalica***	**GCA_000373545.1**	**100.0**	**86.1**
***A. sphaeroplastigenens***	**GCA_002916695.1**	**86.1**	**100.0**
*A. americana*	GCA_900156105.1	24.1	24.3
**ANI (%**)			
**Taxon**	**WGS accession**	***A. alkalica***	***A. sphaeroplastigenens***
***A. alkalica***	**GCA_000373545.1**	**100.0**	**98.6**
***A. sphaeroplastigenens***	**GCA_002916695.1**	**98.6**	**100.0**
*A. americana*	GCA_900156105.1	81.2	81.0

Strains: *A. sphaeroplastigenens* JC133T; *A. alkalica* DSM 8900T; *A. americana* ASpG1T; *A. odontotermitis* JC202T; *A. cellulosivorans* JC227T.

The genome sequence of *A. odontotermitis* JC202T (GenBank accession number GCA_000768055.1) is available, but the quality is poor due to high contamination. A genome sequence of *A. cellulosivorans* JC227T is not available.

Whole genome sequences are publicly available for *A. alkalica* Z-7491^T^ and *A. sphaeroplastigenens* JC133^T^ (GenBank accession numbers GCA_000373545.1 and GCA_002916695.1, respectively). To further explore the phylogenetic relationship between these strains, ortho average nucleotide identity (ANI) [13] and digital DNA–DNA hybridization (dDDH) [14] were calculated between them and their close relatives (Table 1). An orthoANI [15] value of 98.6% was calculated between *A. alkalica* and *A. sphaeroplastigenens*, exceeding the accepted species cut-off boundary for ANI (95–96%). These high levels of relatedness were further supported using the Type (Strain) Genome Server [16], which determined that both genomes belonged to the same species based on a dDDH value of 86.1%, which exceeds the accepted species cut-off boundary (>70.0% [14]) (Table 1). Finally, to further confirm the close taxonomic relationship between both strains, a core gene phylogenomic tree was constructed using the Codon Tree method [17] (Fig. 2). This method selects single-copy PGFams [18]. These aligned proteins and coding DNA sequences from single-copy genes were analysed to build a consensus tree with RAxML [19], and support values were generated using the ‘rapid bootstrapping’ option in RAxML [20]. Also, in our study, *A. alkalica* Z-7491^T^ and *A. sphaeroplastigenens* JC133^T^ formed a monophyletic clade separate from other *Alkalispirochaeta* species and members of other genera (Fig. 2).

**Fig. 2.**
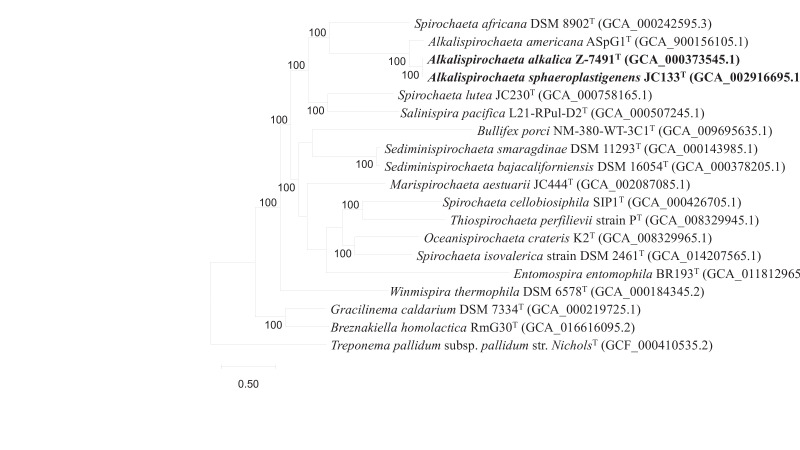
A phylogenetic tree based on the genomes of core-genome phylogenomic tree using a concatenated alignment of 100 single-copy genes showing the relationship among *A. alkalica* Z-7491^T^ and *A. sphaeroplastigenens* JC133^T^ and close relatives. The tree was constructed using Codon Tree. Database accession numbers are given in parentheses. Bar indicates the mean number of substitutions per site, 0.5.

Data regarding the biochemical and physiological comparisons between *A. alkalica* Z-7491^T^ and *A. sphaeroplastigenens* JC133^T^ were previously generated by Reddy *et al*. [4]. Upon comparison, a high level of similarity was observed, with only a few differences. For *A. sphaeroplastigenens* JC133^T^, the production of formate following glucose fermentation and the presence of the polar lipid L2 are observed. In contrast, for *A. alkalica* Z-7491^T^, the presence of polar lipid L5 and hopanoid BHD2 is observed [4]. Given the high level of genetic similarity (Table 1), the presence of near-identical biochemical and physiological characteristics is not unexpected.

Therefore, we argue that, considering the biochemical and physiological similarities, the high phylogenetic congruence observed using 16S rRNA gene (Fig. 1) and core-genome phylogenies (Fig. 2), and the high relatedness based on overall genomic relatedness indices analyses, *A. alkalica* Z-7491^T^ and *A. sphaeroplastigenens* JC133^T^ belong to the same species.

As per the priority of prokaryotic names governed by the International Code of Nomenclature of Prokaryotes [21], when species are united, the earliest validly published name is used for the union. Therefore, *Alkalispirochaeta sphaeroplastigenens* (Vishnuvardhan Reddy *et al*. 2013) Sravanthi *et al*. 2016 is proposed to be a later heterotypic synonym of *Alkalispirochaeta alkalica* (Zhilina *et al*. 1996) Sravanthi *et al*. 2016.

## Emended description of *Alkalispirochaeta alkalica*

The description is as before [2,4] with the following modifications.

The type strain is Z-7491^T^ (=ATCC 700262^T^=DSM 8900^T^) and JC133 (=KCTC 15220=NBRC 109056) is an additional strain. Cells of *A. alkalica* range in size from 0.1 to 0.3 µm in width and 10–20 µm in length. The bacterium grows between 15–40 °C, pH 8.0–10.0, 2.0–12.0% NaCl. The polar lipid L2 is found, but L5 is absent, as is the hopanoid BHD2. The production of formate from glucose by the two strains varies.

The emended DNA G+C content derived from genome sequences is 60.5–60.6 mol%.

## Abbreviations

ANI: average nucleotide identity
dDDH: digital DNA–DNA hybridization
ML: maximum likelihood
